# Paraganglioma of the cauda equina: a tertiary centre experience and scoping review of the current literature

**DOI:** 10.1007/s10143-021-01565-7

**Published:** 2021-05-21

**Authors:** Anan Shtaya, Robert Iorga, Samantha Hettige, Leslie R. Bridges, Simon Stapleton, Francis G. Johnston

**Affiliations:** 1 Neurosciences Research Centre, Molecular and Clinical Sciences Research Institute, St George’s, University of London, London, UK; 2 Atkinson Morley Neurosurgery Centre, St George’s University Hospitals NHS Foundation Trust, London, UK; 3 Wessex Spinal Unit, University Hospital Southampton NHS Foundation Trust, Southampton, UK; 4 Department of Cellular Pathology, St George’s University Hospitals NHS Foundation Trust, London, UK

**Keywords:** Paraganglioma, Cauda equina, Lumbar, Sacral, Intradural

## Abstract

Cauda equina paragangliomas are rare benign extra-adrenal neuroendocrine tumours arising from the neural crest cells associated with autonomic ganglia. These tumours are often mistaken preoperatively for ependymomas or schwannomas. Patients present with axial or radicular pain with or without neurological deficits. Recurrence, secretory features and length of follow-up are controversial. We conducted a retrospective cohort study of paraganglioma through searching a prospectively maintained histopathology database. Patient demographics, presentation, surgery, complications, recurrence, follow-up and outcome between 2004 and 2016 were studied. The primary aim was to collate and describe the current evidence base for recurrence and secretory features of the tumour. The secondary objective was to report outcome and follow-up strategy. A scoping review was performed in accordance with the PRISMA-ScR Checklist. Ten patients were diagnosed (M:F 7:3) with a mean age of 53.6 ± 5.1 (range 34–71 years). MRI scans revealed intradural lumbar enhancing lesions. All patients had complete microsurgical excisions without adjuvant therapy with no recurrence with a mean follow-up of 5.1 ± 1.4 years. Tumours were attached to the filum terminale. Electron microscopic images demonstrated abundant neurosecretory granules with no evidence of catecholamine production. A total of 620 articles were screened and 65 papers (including ours) combining 121 patients (mean age 48.8 and M:F 71:50) were included. The mean follow-up was 3.48 ± 0.46 (range 0.15–23 years). Back pain was the most common symptom (94%). Cure following surgery was achieved in 93% of the patients whilst 7% had recurrence. Total resection likely results in cure without the need for adjuvant therapy or prolonged follow-up. However, in certain situations, the length of follow-up should be determined by the treating surgeon.

## Introduction

Paraganglioma is a rare neuroendocrine extra-adrenal tumour histologically similar to pheochromocytoma but is distinguished by anatomical location [46]. They arise from neural crest-derived cells or paraganglia and may be either sympathetic (secreting catecholamines) or parasympathetic non-secretory lesions [46]. Histologically, they are slow growing with a benign appearance and have been classified as World Health Organization (WHO) grade I tumours [46]. In the central nervous system, the vast majority are located in the jugular glomus, as well as the carotid bodies which are the most common extra-adrenal site [64]. Paragangliomas of the spine as primary tumours are extremely rare with just over 200 cases reported in the English literature so far [43, 67]. The peak incidence is in the 5th decade, with male predominance [20, 26, 67]. The main anatomical spinal site is cauda equina and filum terminale [3, 20, 26, 32, 58, 67]. With such a small number of paraganglioma of the lumbar spine, little is known about this disease.

Complete microsurgical resection remains the first choice. However, on review of the current literature, there is a lack of consensus with regard to clinical management. Firstly, on preoperative imaging, differentiating paraganglioma from other cauda equina tumours is challenging and this might have an impact on the patients’ management. This point has been raised and discussed in a recent review by Honeyman et al. Secondly, some of these tumours are secretory requiring preoperative and intraoperative preparation similar to surgery for carotid body/glomus paragangliomas. Thirdly, there is a lack of consensus about length of follow-up, intraoperative dissemination and recurrence rate especially when complete resection has been achieved.

In an attempt to answer the above questions, we report a consecutive series of surgically treated primary lumbar paragangliomas. We describe the relevant clinical presentation, and radiological and pathological findings together with follow-up, recurrence and outcome. We have also conducted a scoping review of the existing literature. The aim of the scoping review was to search for recurrence following gross total resection and the presence of any secretory features of the tumour or intraoperative complications (hypertensive episodes intraoperatively or whilst in recovery) and to suggest recommendations for follow-up.

## Methods

This retrospective cohort study was registered as an audit with our institutional approval (CADB002408). A database for the case series was created by searching the prospectively maintained neuropathology database for paraganglioma for the period September 2004 to December 2016 inclusive. Patients case notes, electronic records and images were searched. We analysed patient age, sex, comorbidities, presenting symptoms/signs, neurological status at presentation (any neurological deficits), time from presentation to surgery, complications, length of hospital stay and follow-up, recurrence and outcome.

Magnetic resonance imaging (MRI) studies were reviewed. The radiology reports and images were reviewed regarding the level of the lesions, together with their features in term of shape, contour, signal on T1-weighted and T2-weighted MRI sequences and enhancement with gadolinium.

The operative documentations were reviewed for the type of procedure, intraoperative findings and intraoperative complications. Anaesthetic charts were analysed for instability or changes in blood pressure intraoperatively. The recovery charts were reviewed for vital signs especially blood pressure changes.

The histopathological features were analysed from the formal histopathology reports macroscopically (shape, colour, consistency and encapsulation), microscopically (cellular arrangement (Zellballen) and immunohistochemically (necrosis, Ki-67 expression, immunostaining for chromogranin A and synaptophysin, CAM 5.2 (cytokeratin), GFAP and S100 where available). Electron microscopy was reviewed regarding the presence of abundant neurosecretory (dense-core) granules or prominence of rough endoplasmic reticulum and Golgi apparatus.

Statistical analyses were performed using the GraphPad Prism version 8.02 for Windows 10, GraphPad Software, La Jolla, CA, USA.

### Search strategy for scoping review

An extensive literature search was undertaken including PubMed, Google Scholar, Ovid MEDLINE, EMBASE and the Cochrane Central Register of Controlled Trials (CENTRAL). Searches were limited to articles published between 1990 and 2020 inclusive and written in English only. Search terms were charted to subject headings and combined using Boolean operations. The following keywords were used for search: “paraganglioma”, “human”, “spine, “lumbar”, “sacral”, and “cauda equina”. Abstracts of papers found in the literature search were scrutinised independently by two authors (AS and RI) to assess suitability for inclusion. Reference lists from the papers identified in the literature search were manually searched to ascertain other articles suitable for inclusion. The inclusion criterium was any article that described intradural paraganglioma of the cauda equina or lumbo-sacral region. Those with no full text, non-English, animal/cadaveric studies and non-spinal or lumbo-sacral paraganglioma were excluded.

This scoping review has been reported in accordance with the Preferred Reporting Items for Systematic Reviews and Meta-Analysis extension for Scoping Reviews (PRISMA-ScR) [66].

### Outcome

Outcome was assessed in terms of any improvement of preoperative neurological deficits, back pain or sciatic pain by independent clinicians immediately after surgery and 12 months post-operatively. Patients were contacted for a follow-up assessment if they had not attended in person at 12 months post-operatively. Recurrence was assessed on follow-up imaging. Patients who had complete microsurgical resection of the paraganglioma with no recurrence on follow-up were considered cured.

### Synthesis of results

The results in this manuscript are presented as a scoping review, including summary tables, and follow the coming format: patient demographics; presentation and localisation of the tumour; gross total resection and complications; length of follow-up; recurrence and secretory features of the tumour.

## Results

### Case series

**Fig. 1:**
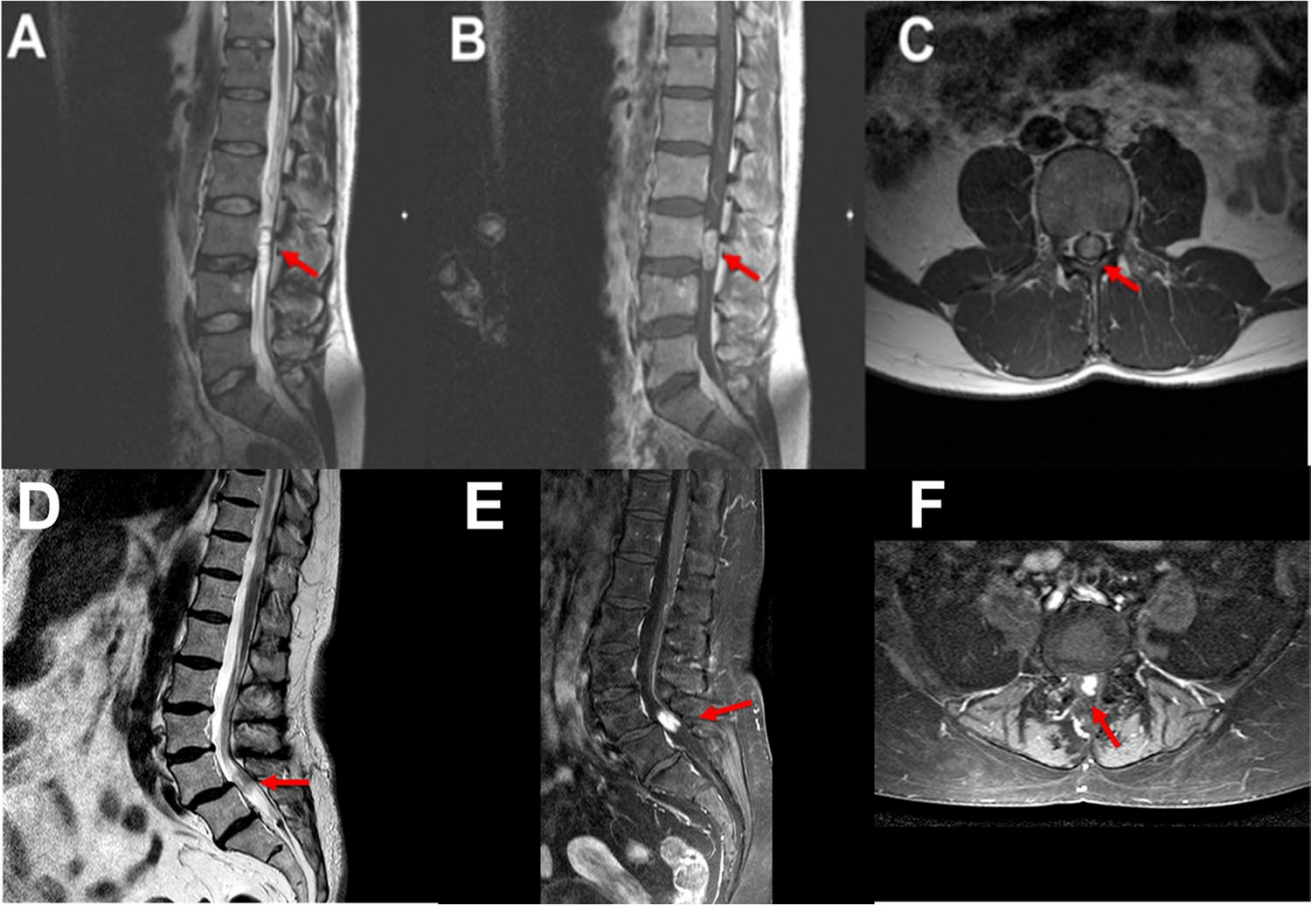
Representative MRI images of paragangliomas. **A–C** Patient number 5 (Table 1) images. **A**) Sagittal T2W image showing the intradural lesion (arrow). **B**) Sagittal post-contrast (Gadolinium) T1W image demonstrating homogenously enhancing lesion (arrow). **C** Axial post-contrast (Gadolinium) T1W image demonstrating an intradural homogenously enhancing lesion (arrow). **D–F** Patient number 9 (Table 1) images. **D**) Sagittal T2W image suspicious of a spinal lesion (arrow). **E**) Sagittal post-contrast (Gadolinium) T1W image demonstrating enhancing lesion (arrow). **F**) Axial post-contrast (Gadolinium) T1W image demonstrating an intradural enhancing lesion (arrow). Please note that in this patient (number 9), the post-contrast MRI was performed at a different date

We report ten patients presenting to our institution with non-syndromic primary lumbar paraganglioma between 2004 and 2016, seven males and three females with a mean age of 53.6 ± 5.1 (range 34–71 years). All patients presented with back pain. The remaining constellation of symptoms are summarised in Table 1. The duration of symptoms ranged from 3 months to 6 years. Magnetic resonance imaging revealed intradural lumbar enhancing lesions. Pre-surgical radiological diagnosis included nerve sheath tumour and ependymoma. None of the cases was reported preoperatively as a paraganglioma. Representative images of two cases are presented in Fig. 1. The mean duration from radiological diagnosis or referral to surgical intervention was 23.1 ± 7.7 days (range 0–70 days). Three patients had surgery within 24 h of admission due to worsening pain and neurological function and they all had complete microsurgical excisions. Two cases underwent intraoperative frozen section pathology results which revealed paraganglioma. All tumours were reported to be of vascular nature and found attached to the filum terminale which was divided at the time of surgery. No intraoperative complications were reported.

**Table 1:** Patient demographics, clinical presentation, MRI localisation, follow-up and outcome

Patient number	Age/sex	Presenting symptoms/signs	Duration	Comorbidities	MRI localisation (intradural)	Time from diagnosis to surgery (days)	Post-surgery complications	Length of hospital stay (days)	Follow-up scans (years), recurrence (Y/N)	Outcome
1	64/M	LBP, perianal numbness and paraesthesia	3 mo	Asthma	L4-5	1	None	8	12 (N)	Symptoms resolved, new LBP, scans showed degenerative changes
2	70/M	LBP	5 mo	Diverticular disease	L2	6	Pseudomeningocele repaired	6	5 (N)	LBP resolved
3	66/M	LBP, left lower limb paraesthesia and mild weakness, absent reflexes and abnormal proprioception	5 mo	DMI, HTN,glucoma,cataract, hypercholestrolemia	L2	52	None	5	2 (N)	LBP resolved, residual mild weakness and abnormal proprioception
4	71/M	LBP and bilateral radiculopathy	6 mo	HTN, glaucoma	L3-4	20	None	4	7 (N)	LBP and radiculopathy resolved
5	34/M	LBP, bilateral radiculopathy, intermittent incontinence	2 y	None	L3-4	70	Post-surgery extradural haematoma evacuated	5	2 (N)	LBP and radiculopathy resolved
6	35/M	LBP and reduced bilateral reflexes	6 y	None	L1-3	44	None	3	2 (N)	LBP resolved
7	36/M	LBP, sudden deterioration at admission with mild lower limbs weakness, perianal anaesthesia and reduced anal tone	18 mo	None	L3-4	1	None	13	2 (N)	LBP, weakness and abnormal sensation resolved
8	48/F	LBP, left radiculopathy, mild left lower limb weakness and paraesthesia	6 mo	None	L4	0	None	6	13 (N)	LBP, radiculopathy and weakness resolved
9	71/F	LBP and left radiculopathy	7 mo	Asthma	L4-5	14	None	7	4 (N)	LBP and radiculopathy resolved
10	41/F	LBP	11 mo	None	L3	23	None	3	2 (N)	LBP resolved

*M* male, *F* female, *LBP* lower back pain, *mo* month, *y* year, *DMI* diabetes mellitus type I, *HTN* hypertension, *L* lumbar, *Y/N* yes/no

**Fig. 2:**
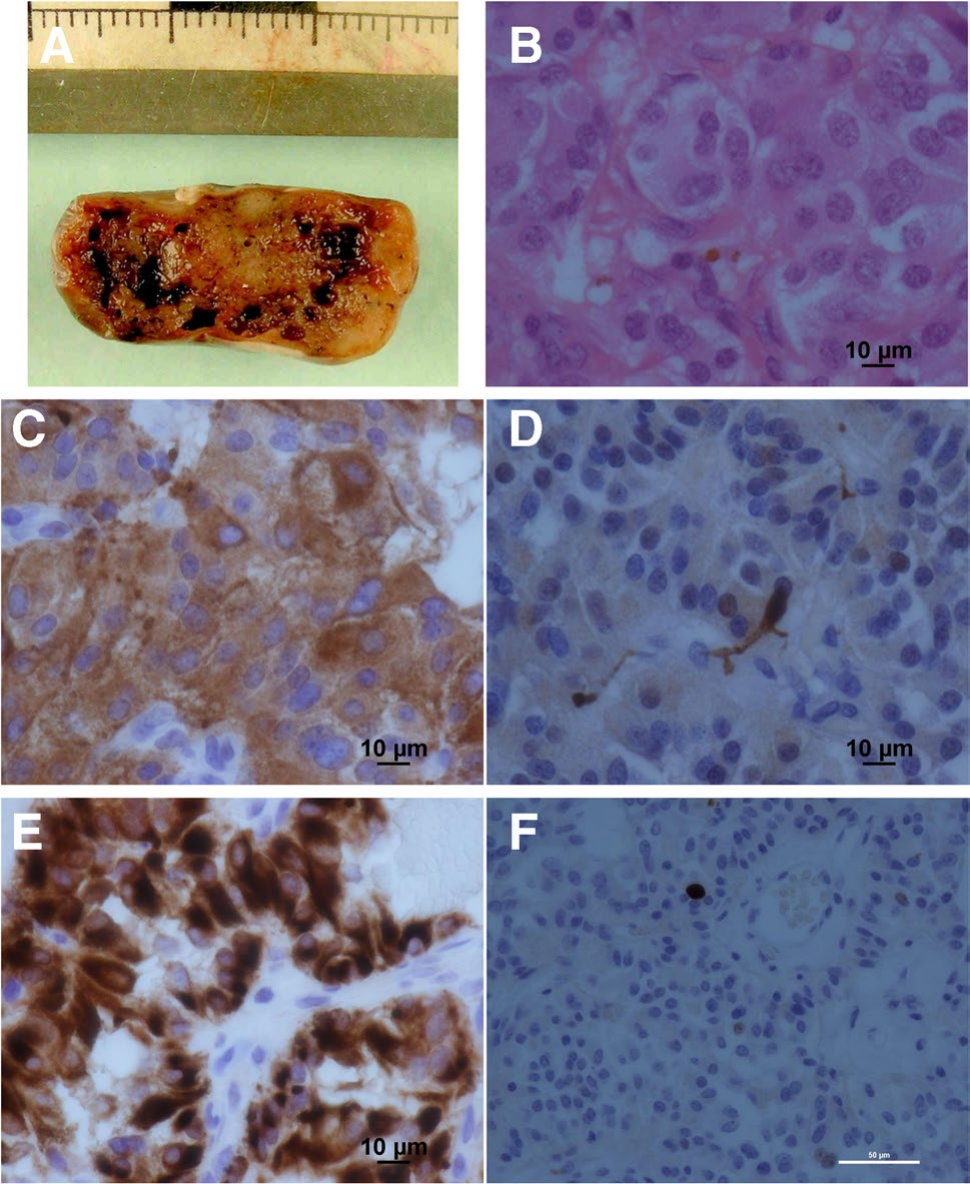
Histopathology paraganglioma samples. **A**) Macro specimen — tissue filled with blood cysts. **B**) Characteristic rounded groups of cells, polygonal to oval and are arranged in distinctive cell balls called Zellballen. Haematoxylin and eosin, scale bar = 10 microns.** C**) Diffuse immunostaining for neurosecretory granules synaptophysin, scale bar = 10 microns. **D**) S100-positive sustentacular cells. Immunostain for S100 and negative for GFAP, scale bar = 10 microns. **E**) The tumour cells contain cytokeratin. Immunostain for CAM5.2, scale bar = 10 microns. **F**) There is a low proliferation rate. Immunostain for Ki-67, scale bar = 50 microns

**Fig. 3:**
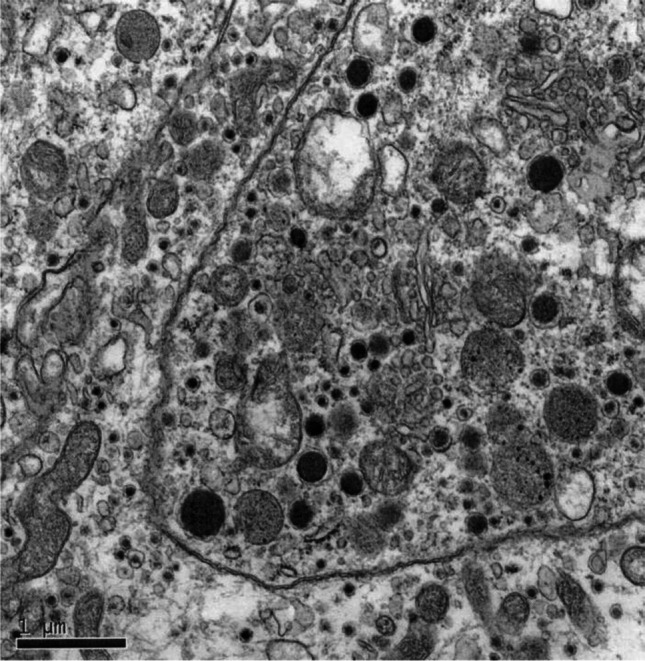
Electron micrograph of paraganglioma. Electron micrograph showing dense-core neurosecretory granules and prominent rough endoplasmic reticulum and Golgi apparatus. Imaged on a Hitachi 7100 EM at magnification × 26,100; scale bar = 1 micron

Histopathological and electron microscopic studies were carried out and confirmed a classic “Zellballen” pattern (Fig. 2), the presence of dense-core neurosecretory granules, prominent rough endoplasmic reticulum and Golgi apparatus (Fig. 3). The immunohistochemical studies confirmed that the tumours had low proliferation rates (Ki67 cell counts (< 3%) and profiles compatible with paraganglioma WHO grade 1 (Fig. 2).

Anaesthetic and recovery charts were reviewed and there was no evidence of either hypertensive episodes or hemodynamic instability. Patients received 24–48 h of dexamethasone (4 or 8 mg twice a day) as per the treating surgeon’s instruction. The mean length of hospital stay was 6 days (range 3–13 days). All patients, except one who required rehabilitation, were discharged home. None of the patients received any adjuvant therapy and we found no recurrence of the tumour after a mean follow-up of 5.1 ± 1.4 years. All patients reported improvements in their back pain, radiculopathy and neurological function (Table 1).

We experienced low complications. One patient developed mild weakness, worsening back pain and sciatica secondary to an epidural haematoma that was evacuated promptly with no adverse effects. Another patient developed a pseudomeningocele that required surgical repair.

### Scoping review

#### Patient demographics and presentation

**Fig. 4:**
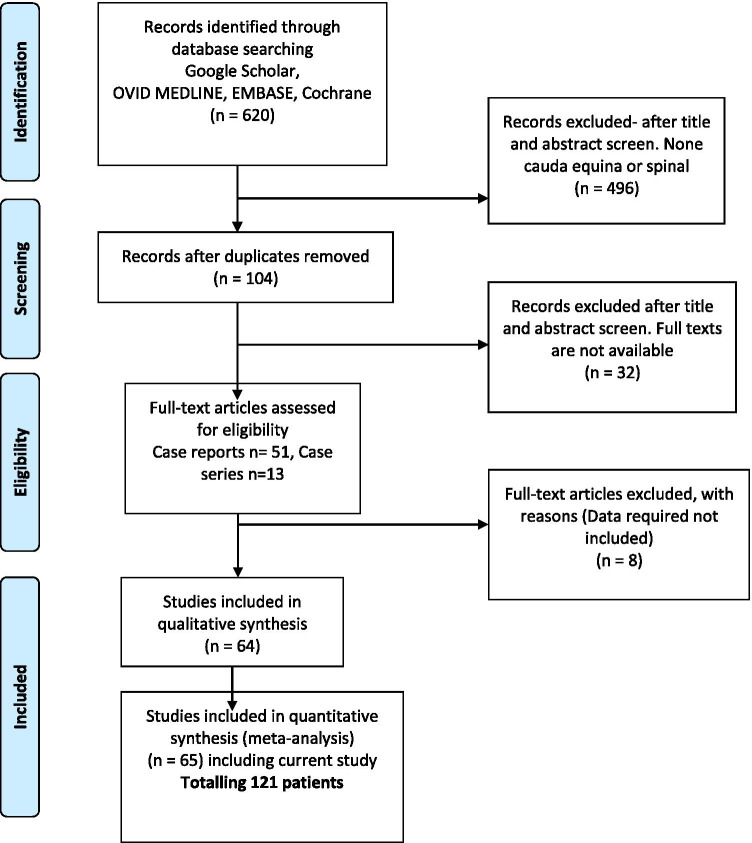
PRISMA flow diagram of Google scholar, PubMed, Ovid, Medline, Embase and Cochrane between January 1990 and July 2020

The review search revealed 620 articles where the title and abstracts were screened and 65 papers (including ours) combining 121 patients (Fig. 4). We have included 51 case reports [1, 2, 5–14, 16–19, 22, 24, 25, 27, 28, 31, 34, 37–41, 44, 45, 47, 48, 50, 52, 53, 55–57, 59, 62, 63, 69–77] and 14 case series [4, 15, 20, 21, 30, 49, 51, 58, 60, 61, 67, 68, 78] (Tables 2 and 3). The mean patients’ age was 48.8 ± 1.2 (range 17–75 years).

**Table 2:** Review case reports. Patient demographics, clinical presentation, MRI localisation, follow-up and outcome

Article	Age	Sex	Back pain/sciatica	Sensory changes	Weakness	Bowel/bladder problems	Reduced reflexes	Others	Location of the tumour	Other images description	GTR (Y/N)	Complications	Length of follow-up	Recurrence	Secretory (yes or no)
			Presentations												
Djindjian, M. et al. 1990	36	M	Yes	Yes	Yes	Yes	Yes	-	L1-5		Yes	No	15 m	No	No
Iliya, A. R. et al. 1991	35	M	No	Yes	Yes	Yes	No	-	L2-4		Yes	No	No f/u	-	No
Caccamo, D. V. et al. 1992	31	F	Yes	No	No	No	Yes	-	L5-S2		Yes	No	No f/u	-	No
Mylonas, C. 1992	62	M	Yes	Yes	Yes	No	No	-	Conus		Yes	No	6 m	No	No
Hardten, D. R. et al. 1992	56	M	Yes	Yes	No	No	No	Papilloedema	L3		-	-	No f/u	-	No
Aggarwal, S. et al. 1993	44	F	Yes	No	Yes	No	Yes	-	Conus to S1		Yes	No	No f/u	-	No
Boukobza, M. et al. 1993	61	F	Yes	No	No	No	No	-	L4-S1		Yes	No	No f/u	-	No
Wester, D. J. et al. 1993	61	F	Yes	Yes	Yes	No	Yes	-	L2-3		Yes	No	No f/u	-	No
Steel, T. R. et al. 1994	50	F	Yes	Yes	Yes	No	No	-	L1-3	Syringomyelia	Yes	No	18 m	No	No
Roche, P. H. et al. 1996	57	F	Yes	No	No	Yes	No	-	L5-S1		No	No	4 y	Yes	No
Faro, S. H. et al. 1997	46	F	Yes	Yes	Yes	No	Yes	-	Conus		Yes	No	1y	No	No
Ashkenazi, E. et al. 1998	40	M	Yes	No	No	No	No	Papilloedema	L4-5		Yes	CSF leak	3 m	No	No
Paleologos, T. S. et al. 1998	62	F	Yes	Yes	No	Yes	Yes	Headaches/papilloedema	T12-L3		Yes	No	2 y	No	No
Herman, M. et al. 1998	46	M	Yes	No	No	No	Yes	-	L2-4		Yes	No	6 m	No	No
Sharma, A. et al. 1998	60	M	Yes	No	No	No	No	Confusion/ataxia/hearing impairment	L4-5		Yes	No	No f/u	-	No
Wang, Y. F. et al. 2000	45	M	Yes	No	No	No	No	-	L3		Yes	No	No f/u	-	No
Lalloo, S. T. et al. 2001	36	M	Yes	Yes	Yes	No	No	-	L2-4		No	Yes	No f/u	-	No
Parthiban, J. K. B. C. et al. 2004	29	F	Yes	No	No	No	No	-	L3	Hourglass configuration	Yes	No	No f/u	-	No
Sankhla, S. et al. 2004	29	M	Yes	No	No	No	No	Papilloedema	L2		Yes	No	No f/u	-	No
Bannykh, S. et al. 2005	50	F	No	Yes	Yes	No	Yes	Papilloedema	L3-S1		Yes	No	No f/u	-	No
Matschke, J. et al. 2005	63	F	Yes	No	No	Yes	No	-	L2-3		Yes	No	No f/u	-	No
Pytel, P. et al. 2005	74	F	Yes	No	No	No	No	-	-		-	-	-	-	-
Bozkurt, G. et al. 2005	52	M	Yes	Yes	No	No	No			“Silk cocoon” appearance on spinal angiography	Yes	No	No f/u	-	No
Slowinski, J. et al. 2005	46	F	Yes	No	No	No	No	-	L3		Yes	No	No f/u	-	No
Walsh, J. C. et al. 2005	26	M	Yes	Yes	Yes	Yes	Yes	-	L2-4		Yes	No	2 y	No	No
Warrier, S. et al. 2006	54	M	Yes	No	No	No	No	-	L3		Yes	No	4 y	Yes	No
Li, P. et al. 2007	36	F	Yes	Yes	Yes	No	Yes	-	L3		Yes	No	1 y	No	No
Vural, M. et al. 2008	17	M	Yes	No	Yes	No	Yes	-	L4		-	-	-	-	-
Marcol, W. et al. 2009	43	M	Yes	No	No	Yes	Yes	-	L2-3		Yes	No	11 m	No	No
Erban, T. et al. 2010	38	F	Yes	No	No	No	No	-	L3		Yes	No	15 m	No	No
Rhee, H. Y. et al. 2010	70	M	No	No	Yes	Yes	No	NPH	T12-L1		Yes	No	No f/u	-	No
Mahalingashetti, P. B. et al. 2012	60	F	Yes	No	No	No	No	-	L3-4		Yes	No	1 y	No	No
Agrawal, V. et al. 2012	50	M	Yes	Yes	Yes	Yes	No	-	T12-L2	Significant scalloping of post margins of vertebral bodies	No	Yes	Pt died	-	Yes
Adriani, K. S. et al. 2012	34	M	No	No	No	No	No	Papilloedema	L5-S1		Yes	No	No f/u	-	No
Hong, J. Y. et al. 2012	47	F	Yes	Yes	No	No	No	-	L2-4		Yes	No	2 y	No	No
Midi, A. et al. 2012	38	F	Yes	No	No	No	No	-	L3-4		Yes	No	15 m	No	No
Undabeitia-Huertas, J. et al. 2013	47	F	Yes	Yes	Yes	Yes	No	-	L2-4		Yes	No	No f/u	-	No
Bhatia, R. et al. 2013	33	M	Yes	Yes	Yes	No	No	-	L5-S1		Yes	No	1 y	No	No
Bush, K. et al. 2014	72	M	No	No	No	No	No	Headaches/papilloedema	L5		Yes	No	No f/u	-	No
Sable, M. N. et al. 2014	58	M	Yes	No	Yes	No	No	-	L2		Yes	No	16 m	No	No
Corinaldesi, R. et al. 2015	33	F	Yes	No	No	No	No	-	L3		Yes	No	3 y	No	No
Dillard-Cannon, E. et al. 2016	32	M	Yes	No	No	No	No	-	L3		Yes	No	No f/u	-	No
Chou, S. C. et al. 2016	58	M	Yes	No	Yes	Yes	No	-	-		No	No	No f/u	-	No
Hilmani, S. et al. 2016	74	F	Yes	No	Yes	Yes	Yes	-	L3-4		Yes	No	No f/u	-	No
Yaldiz, C. et al. 2016	64	F	Yes	Yes	Yes	Yes	Yes	-	L3		Yes	No	No f/u	-	No
Satyarthee, G. D. et al. 2017	40	F	Yes	No	No	No	No	-	L3		Yes	No	No f/u	-	No
Murrone, D. et al. 2017	56	M	Yes	Yes	Yes	Yes	Yes	-	L1-2		Yes	No	1 y	No	No
Wang, Z. H. et al. 2018	36	M	Yes	No	No	No	No	-	L1		Yes	-	6 mo	No	No
Mendez, J. C. et al. 2019	75	M	Yes	Yes	No	Yes	No	-	L3-4		Yes	No	24 mo	No	No
Vats, A. et al. 2019	63	F	Yes	No	No	Yes	No	NPH	L5-S1		Yes	No	3 mo	No	No

*M* male, *F* female, *LBP* lower back pain, *m* month, *y* year, *GTR* gross total resection, *NPH* normal pressure hydrocephalus, *T* thoracic, *L* lumbar, *S* sacral, *f/u* follow-up, *CSF* cerebrospinal fluid, *Pt* patient, *Y/N* yes/no

**Table 3:** Review case series. Patient demographics, clinical presentation, MRI localisation, follow-up and outcome

Article	Age	Sex	Back pain/sciatica	Sensory changes	Weakness	Bowel/bladder problems	Reduced reflexes	Others	Location of the tumour	Other images description	GTR (Y/N)	Complications	Length of follow-up	Recurrence	Secretory (yes or no)
			Presentations												
Pigott, T. J. D. et al. 1990	37	F	Yes	No	Yes	No	No/brisk	-	Conus		Yes	No	-	-	No
	36	M	Yes	Yes	No	No	No	-	Cauda equina		Yes	No	6 m	No	No
	53	M	Yes	No	No	No	Yes	-	Cauda equina		Yes	No	6 m	No	No
Raftopoulos, C. et al. 1990	47	M	No	Yes	Yes	Yes	Yes	-	L3		Yes	No	17 m	No	No
	33	M	Yes	No	No	No	No	-	L2-3		Yes	No	8 y	Yes	No
Aghakhani, N. et al. 1999	67	F	Yes	No	No	No	No	-	L3-4		Yes	No	6 m	No	No
	34	M	Yes	Yes	Yes	Yes	No	-	L3-5		Yes	No	3 m	No	No
Gelabert-Gonzalez, M. 2005	62	F	Yes	Yes	No	No	Yes	-	L3-4		Yes	No	5 y	No	Yes
	49	M	Yes	No	No	No	No	-	L5		Yes	No	2 y	No	No
Yang, S. Y. et al. 2005	49	M	Yes	No	No	No	No	-	L3		Yes	No	33 m	No	No
	63	F	Yes	No	No	No	No	-	L4-5		Yes	No	40 m	No	No
	71	F	Yes	No	No	No	No	-	L4		Yes	No	24 m	No	No
	52	F	Yes	No	Yes	No	No	-	L3		Yes	No	71 m	No	No
Singh, N. G. et al. 2005	22	M	Yes	No	No	No	No	-	L2-3		Yes	No	9 y	No	No
	28	M	Yes	No	No	No	No	-	Conus		Yes	No	5 y	No	No
	60	M	Yes	Yes	No	No	No	-	L4-5		Yes	No	3 y	No	No
	50	M	Yes	Yes	No	Yes	No	-	L3		Yes	No	2 y	No	No
	35	F	Yes	Yes	No	Yes	No	-	L2		Yes	No	2 y	No	No
	50	M	Yes	Yes	No	No	No	-	L2-3		Yes	No	1 y	Yes	No
	33	F	No	No	No	No	No	-	L3-4		Yes	No	6 m	No	No
Demircivi Ozer, F. et al. 2010	75	M	Yes	Yes	Yes	Yes	No	-	L3-L4		Yes	No	No f/u	-	No
	70	M	Yes	No	No	No	No	-	L1-2		Yes	Yes-haematoma	No f/u	-	No
	50	M	Yes	Yes	No	No	No	-	L1-4		Yes	No	No f/u	-	No
*Kimura, N. et al. 2011	50	M	Yes	No	No	No	No	-	L4		Yes	No	2 m	No	No
	54	M	Yes	No	No	No	No	Headaches/vomiting	S1		Yes	No	2 y	No	No
Simsek, M, et al. 2015	36	M	Yes	No	No	No	No	-	L2		Yes	No	8 m	No	No
	40	M	Yes	Yes	No	No	No	-	L4		Yes	No	6 m	No	No
Turkkan, A. et al. 2019	47	F	Yes	No	No	No	No	-	L4-5		Yes	No	No f/u	-	No
	48	F	Yes	No	No	No	No	-	L5		Yes	No	No f/u	-	No
Seidou, F. et al. 2020	54	M	No	No	No	No	No	-	-		-	-	-	-	-
	63	F	No	No	No	Yes	No	-	L4-5		-	-	-	-	-
	46	M	Yes	No	No	No	No	-	L1-2		-	-	-	-	-
	71	M	Yes	No	No	No	No	-	S1-2		-	-	-	-	-
	41	M	Yes	No	Yes	No	No	-	L3-4		-	-	-	-	-
	45	M	Yes	No	No	No	No	-	L1-2		-	-	-	-	-
	50	M	Yes	No	No	No	No	-	L1-2		-	-	-	-	-
	62	M	Yes	No	No	No	No	-	L2-3		-	-	-	-	-
	48	F	Yes	No	No	No	No	-	L4-5		-	-	-	-	-
Tuleasca, C. et al. 2020	34	M	Yes	No	No	No	No	-	T12-L1		Yes	-	23 y	No	No
	35	F	Yes	No	No	No	No	-	T12-L1		Yes	-	13 y	No	No
	37	F	Yes	No	No	No	No	-	Cauda equina dissemination		No	-	11 y	Yes	No
**	44	F	Yes	No	No	No	No	-	T3		No	-	4 y	Yes	No
	48	F	Yes	No	No	No	No	-	L1-S2		No	-	30 m	No	No
	39	F	Yes	No	No	No	No	-	L1-3		Yes	-	12 y	No	No
	36	M	Yes	No	Yes	No	No	-	L3-4		Yes	-	7 y	No	No
	30	M	Yes	No	No	No	No	-	T12-L1		Yes	-	2 y	No	No
	53	M	Yes	No	No	No	No	-	L2		Yes	-	1 y	No	No
Fiorini, F. et al. 2020	53	M	Yes	Yes	No	No	No	-	L2-3		-	-	1.6 m	-	-
	42	F	Yes	No	No	No	No	-	L3-4		-	-	5 days	-	-
	33	M	Yes	No	Yes	Yes	No	-	L4		-	-	5.2 m	-	-
	60	M	Yes	No	No	No	No	-	L3-4		-	-	6.6 m	-	-
	62	M	Yes	No	No	No	No	-	L4		-	-	1.4 y	-	-
	41	F	Yes	No	No	No	No	-	L4		-	-	1.7 y	-	-
	53	F	Yes	No	No	No	No	-	L2-3		-	-	5.5 y	-	-
	38	M	Yes	Yes	No	Yes	No	-	L1-2		-	-	5.4 y	-	-
	34	F	Yes	No	No	No	No	-	L2		-	-	9.6 y	-	-
	56	F	Yes	No	Yes	No	No	-	L3		-	-	3.6 y	-	-
	68	M	Yes	No	No	No	No	-	L3		-	-	2.9 y	-	-
	66	M	Yes	No	Yes	Yes	No	-	L4		-	-	12 y	-	-
	55	M	Yes	Yes	Yes	Yes	No	-	L3-4		-	-	6.9 y	-	-

*M* male, *F* female, *LBP* lower back pain, *m* month, *y* year, *GTR* gross total resection, *T* thoracic, *L* lumbar, *S* sacral, *f/u* follow-up, *Pt* patient, *Y/N* yes/no

^**^Excluded as this case is thoracic

One hundred and fourteen patients (94%) presented with back pain, and this was the most common symptom. Sensory changes were reported in 34 patients (34%), and in 32 (26%) patients, lower limb weakness was encountered. Bowel and bladder disturbances were documented in 27 patients (22%), and 18 (15%) patients had reduced lower limbs reflexes. Interestingly, eight (6.6%) patients presented with papilloedema and two patients were diagnosed with normal pressure hydrocephalus. The detailed patients’ presentations are described in Tables 2 and 3.

#### Localisation, surgery and complications

The majority of the tumours were located at the lumbar L1 level or below (Tables 2 and 3). One case was reported in the thoracic spine (at T3) [67]. Although this case was part of one of the case series in the review, we excluded it from the analysis as it is not in the cauda equina region. Gross total resection was reported in 80 cases (66%) and subtotal resection in five cases (4%). There was no data regarding resection in 36 cases (30%). Complications were reported in six cases, such as CSF leak and haematoma (Tables 2 and 3). Two of these are also included in our local series (Table 1).

#### Follow-up, recurrence and secretory features

The mean follow-up interval was 3.48 ± 0.46 (range 0.15–23 years) in a cohort of 79 cases (including ours).

Data concerning recurrence status was reported in 75 cases (63%). Following surgery, 93% had no tumour recurrence, whilst recurrence was reported in 5 cases (7%) [51, 53, 61, 67, 75] (Table 4). Two patients had subtotal resection [53, 67], one patient had multiple other sacral lesions that had grown requiring surgical intervention [75] and the two other patients had no initial post-surgery MRI scan to confirm the total resection of the tumour [51, 61].

**Table 4:** Case with recurrence. Patient demographics, clinical presentation, MRI localisation, follow-up and outcome

Article	Age/sex	Back pain/sciatica	Sensory changes	Weakness	Bowel/bladder problems	Reduced reflexes	Location of the tumour	GTR (Y/N)	Complications	Length of follow-up	Secretory (yes or no)	Comments
Raftopoulos, C. et al. 1990	33/M	Yes	No	No	No	No	L2-3	Yes	No	8 y	No	No initial post-surgery MRI
Roche, P. H. et al. 1996	57/F	Yes	No	No	Yes	No	L5-S1	N	No	4 y	No	Subtotal resection with body metastasis
Warrier, S. et al. 2006	54/M	Yes	No	No	No	No	L3	Yes	No	4 y	No	Growth of other lesions rather than recurrence of the resected one
Singh, N. G. et al. 2005	50/M	Yes	Yes	No	No	No	L2-3	Yes	No	1 y	No	No initial post-surgery MRI
Tuleasca, C. et al. 2020	37/F	Yes	No	No	No	No	Cauda equina dissemination	No	-	11 y	No	Subtotal resection

*M* male, *F* female, *LBP* lower back pain, *y* year, *GTR* gross total resection, *T* thoracic, *L* lumbar, *S* sacral, *Y/N* yes/no

Data about secretory features was available for 96 cases (79%), of which only two cases (2%) were reported to be secretory [5, 21].

The first case was a 62-year-old woman who was also found to be hypertensive [21]. During surgery, she had tachycardia and a rise in blood pressure when manipulating the tumour [21]. The tumour was vascular and clipping of the tumour pedicle facilitated the resection.

The second case with secretory features was a 50-year-old man who was also known to have hypertension, and the MRI revealed a large T12-L2 intradural enhanced lesion with scalloping of the vertebrae [5]. During surgery, the patient had a rise in blood pressure and the tumour was partially resected. Post-operatively, he had flushing all over the body, especially over the face and chest region, palpitations, dysphagia and uncontrolled blood pressure. In spite of intensive care management, he collapsed with haematemesis and died [5].

## Discussion

Primary paragangliomas of the spine are rare, slowly growing, benign intradural extra medullary tumours [20, 67].They are most commonly located in the cauda equina and filum terminale [4, 20, 67] representing approximately up to 3.5% of the cauda equina lesions [42]. They are classified as World Health Organization (WHO) grade I tumours, due to their indolent behaviour and histologically benign appearance.

The most common presenting symptom in a patient with a lumbo-sacral paraganglioma is lower back pain with radiculopathy [7, 20, 58, 67]. Lower back pain was reported in 94% of the cases in our review. The lesion often occupies the whole diameter of the spinal canal; yet, it is rare for it to cause a cauda equina syndrome until very late. Severe/permanent sensory and motor deficits are unusual, and incontinence of urine and faeces rarer still. This was evident in our review (Tables 2 and 3). Commonly, presentation and diagnosis are delayed by months to years as shown in our case series (Table 1) which reflects the nonspecific nature of the symptoms. Due to their slow growth, these lesions are less likely to cause cauda equina syndrome. In our case series, only three patients (cases number 1, 7 and 8 in our series (Table 1)) underwent surgery within 24 h due to their presentation as possible cauda equina syndrome and deteriorating neurological function. Cases 1 and 7 had perianal numbness and case 8 had progressive weakness.

The diagnostic procedure of choice for an intradural lesion is MRI, although it should be noted that the MRI findings are nonspecific for these lesions. Paragangliomas are usually isointense to spinal cord on T1-weighted images, hyperintense on T2-weighted images and enhance with gadolinium [15, 54, 58, 67]. Whilst MRI gives accurate anatomical information regarding intradural cauda equina lesions, the differential diagnosis includes schwannoma, ependymoma, meningioma or solitary metastasis [23] and remains difficult to diagnose paraganglioma preoperatively based solely on the MRI findings. Indeed, a recent review described that preoperative radiological diagnosis of these rare tumours can be challenging [26]. In agreement, certainly, in our case series, none of the cases was reported as paraganglioma before surgery.

An important question we aimed to explore in our study was as to whether cauda equina paragangliomas have a secretory function or not. These patients usually lack the classical clinical triad of headache, diaphoresis and tachycardia (with or without palpitations) which is usually seen in cases of pheochromocytoma (however, the secretory function can be addressed preoperatively by investigating the patients for catecholamines). Cauda equina paragangliomas are rare and therefore unlikely to be investigated for catecholamines preoperatively.

The other clue to suggest that the tumour may be a secretory paraganglioma is an intraoperative surge of catecholamines that causes hypertension and tachycardia. This was not encountered in our case series. Searching the literature, we found a handful of cases (including in other spinal locations) with secretory functions and mainly diagnosed intraoperatively [5, 21, 29, 65, 79].

Although the electron microscopic appearances in our case series revealed the presence of secretory granules (Fig. 3), they had no preoperative, intraoperative or post-operative symptomatology to suggest catecholamine release. This suggests that these lesions are in fact non-functional tumours. Furthermore, unlike pheochromocytoma, sympathetic paragangliomas rarely secrete adrenaline, since the enzyme needed to convert norepinephrine to adrenaline (phenylethylamine *N*-methyltransferase) is expressed exclusively in the adrenal glands [33].

Our series and literature search suggest that it is highly unlikely that an intradural cauda equina paraganglioma behaves like pheochromocytoma and perhaps should not be treated as such. If hypertension and tachycardia are encountered intraoperatively, then clipping the tumour pedicle is helpful in controlling catecholamine surge as described previously [21].

Paragangliomas are indolent WHO grade I lesions [35, 36]. They are usually soft, red, vascular and well-circumscribed masses (Fig. 2A), usually arising from the filum terminale and less commonly from a nerve root. They may be attached to the conus medullaris or to the adjacent nerve roots. As such, microsurgical separation may be difficult. Most commonly the tumour is well-encapsulated and complete resection is accomplished. In our case series, all tumours were found originating from the filum terminale. The primary treatment for cauda equina paraganglioma is complete microsurgical resection which should result in patients’ cure. Similar to other recent large case series [20], we had no recurrence after complete surgical resection after a mean follow-up of 5.1 ± 1.4 years. Overall, we found a 7% recurrence rate in the literature over the years, and in some cases, this was part of an aggressive and metastatic process [53]. Other cases had subtotal resection of the tumour (Table 4), and no initial post-surgery MRI was performed to confirm complete resection of the tumour in the rest (Table 4). We acknowledge that a very small number of recurrences has been reported in the literature [26], and others may recommend longer follow-up period. Following this study, our most recent practice is to follow up the patients in clinic and to perform an initial follow-up scan usually in 3–6 months and discharge if complete microsurgical resection is confirmed.

Although the vast majority of these cases do not recur following complete microsurgical resection that is confirmed on a follow-up MRI scan, the length of follow-up should be on a case by case ground and at the discretion of the treating surgeon.

## Limitations

These results come with the limitations of a retrospective study. The number of cases we report is small. We attempted to increase the number of cauda equina paraganglioma cases by performing a scoping-analysis; however, many of those are case reports and the rest are small case series. This is challenging perhaps due to the nature of the pathology studied.

## Conclusions

Cauda equina paraganglioma is a rare, benign but treatable pathology with very good outcomes. Very rarely they release catecholamines, in our review, we found only two cases. Total microsurgical resection likely provides cure for the patients without the need for adjuvant therapy or prolonged follow-up. However, in certain situations, the length of follow-up should be determined by the treating surgeon.

## Data Availability

Researchers can apply for access to anonymized data from the present study for well-defined research questions. Please contact the corresponding author.
